# Direct-to-Consumer Academic Telemedicine

**DOI:** 10.1089/tmr.2022.0001

**Published:** 2022-03-09

**Authors:** Joshua W. Elder, Daniel Stein, Tamara L. Scott

**Affiliations:** ^1^ UC Davis Health, Sacramento, California, USA.; ^2^ Department of Emergency Medicine, University of California Davis School of Medicine, Sacramento, California, USA.; ^3^ Department of Innovation Technology, UC Davis Health, Sacramento, California, USA.; ^4^ Department of Ambulatory Practice Innovation, UC Davis Health, Sacramento, California, USA.

**Keywords:** direct-to-consumer, on demand, telehealth, telemedicine

## Abstract

Over the past 2 years, telemedicine has skyrocketed as COVID-19 propelled innovation and implementation at unparalleled rates. Within the UC Davis academic health system, a new paradigm for telemedicine emerged: direct-to-consumer telemedicine. The video-based telemedicine program has become the largest of its kind in California and is staffed by 80 providers (MDs, APPs) across five clinical departments/groups (primary care practice group, family and community medicine department, emergency medicine department, the nursing department, and the physical medicine and rehabilitation department). September 2021 marked the 1-year anniversary of a journey that has opened access, improved coordination, and become a workforce engine for our evolving virtual health infrastructure.

Telemedicine visits have skyrocketed during the COVID-19 pandemic and patient experience and preference for the alternative care setting are beginning to become better understood.^1^ Direct-to-consumer (DTC) telemedicine is a rapidly growing sector of the telemedicine industry but most of the publications to date have focused on corporate models and has not focused on academic or integrated network DTC services.^2^

Our academic DTC telemedicine program (Express Care) is a combination of technology, providers, and coordination of care. It began as a fully integrated platform for our tertiary medical center to complement in-person care. Our integrated platform consists of an EMR (Epic Systems Corporation) and a synchronous video platform (ExtendedCare Solutions). Epic provides the patient web portal (MyChart), patient registration, patient messaging/queuing, documentation, coding, and billing functionality. ExtendedCare provides two-way synchronous video, patient testing, waiting room experience, and interpreter integration, and video telemetry.

Our health system was attempting to adapt to a variety of patient access needs, clinical workforce shortages, and, at the time, an evolving threat of COVID-19. The technology connects patients and providers, nurses facilitate follow-up testing, a patient contact center routes referrals, and a help desk improves the real-time patient–provider connection and experience. In summary, Express Care is a fully functioning ambulatory virtual clinic enabling testing and coordination—laboratories, imaging, and referrals.

Although Express Care launched in September of 2020, the infrastructure was initially built in late 2019 when the health system decided to in-source its DTC telemedicine platform instead of continuing to outsource to a corporate staffing model. The timing of COVID-19 and the patient and health system preference for virtual care shifted the program from a pilot to a critical community health care resource, and patient experience surveys quickly highlighted how the program was a preferred clinical setting for patients.

Press Ganey surveys summarized the following at the end of year 1: 80.7% recommend our practice to others, 82.4% were satisfied with ease of communication platform over video, and there were no outlining characteristics that were negative. Initially the service was only open to patients who had an existing medical record with our health system.

Throughout the first year, our operation and technology teams have collaborated to find ways to enhance equity and access to the platform. From the beginning, all contracted insurance payers were accepted and the out-of-pocket expense for patients is the same as the out-of-pocket expense from an in-person ambulatory visit. Figure 1 outlines the number of visits by level of service. Figure 2 outlines the distribution of primary payers. In February 2021, our on-demand language interpretation was fully integrated into visits based on patients preferred language.

**FIG. 1.**
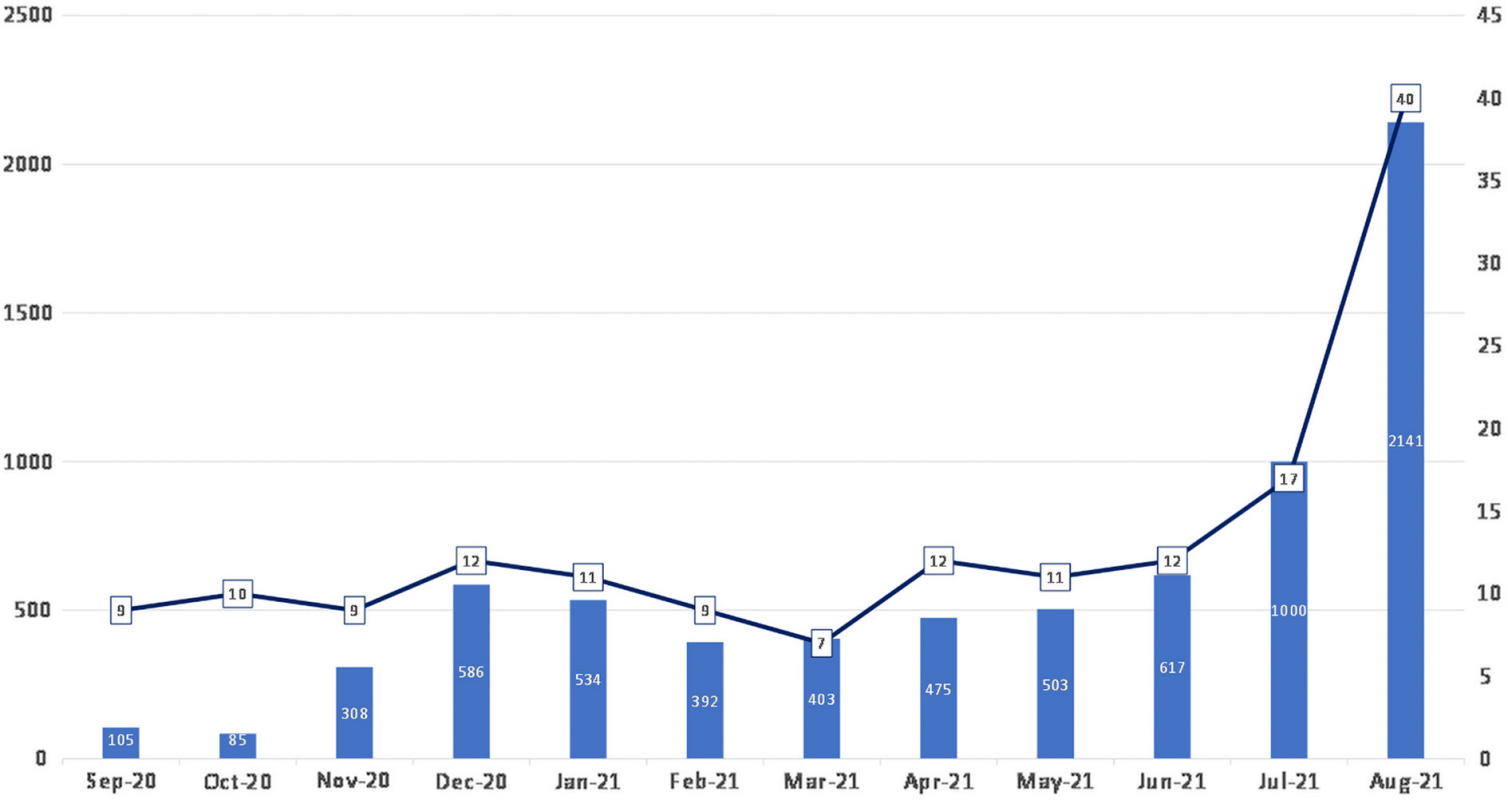
Number of visits by level of service. Vertical axis = number of visits. Horizontal axis = level of service.

**FIG. 2.**
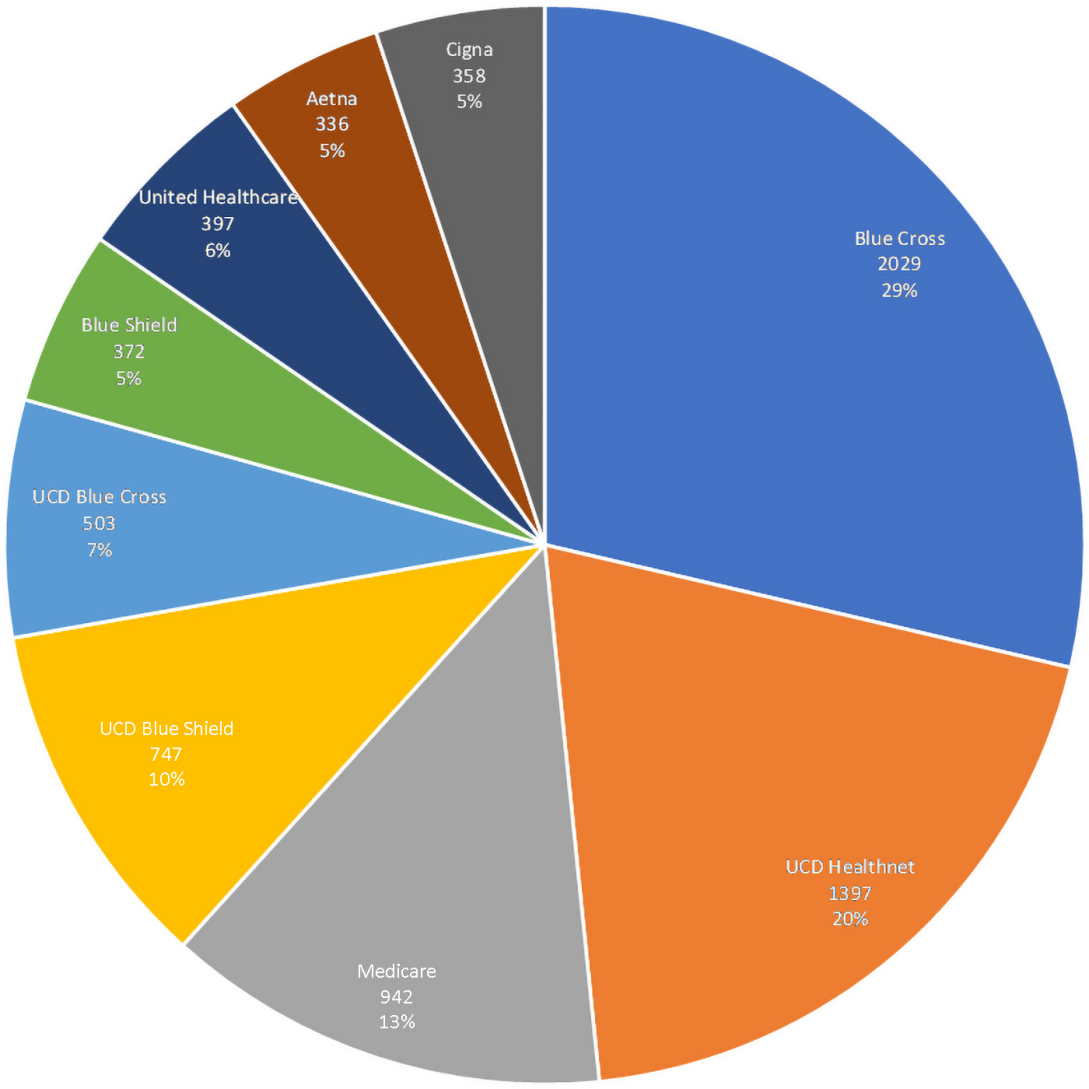
Number of visits by primary payer. Pie chart highlighting the percentage of visits by primary payer.

Express Guest was created in March of 2021 that allows for any adult in California to access the service regardless of whether they had an established relationship with the health system. Recently, Express Guest proxy access was created in August of 2021 to serve patients under the age of 18 years who are not yet affiliated with UC Davis Health and desire virtual medical treatment. Pediatric access, for established patients, however, has been available since the inception of the program. Figure 3 outlines the monthly volume and the average wait time. For the first year of the service, the mean wait time was 11.9 min (SD 7.9, range 4–48).

**FIG. 3.**
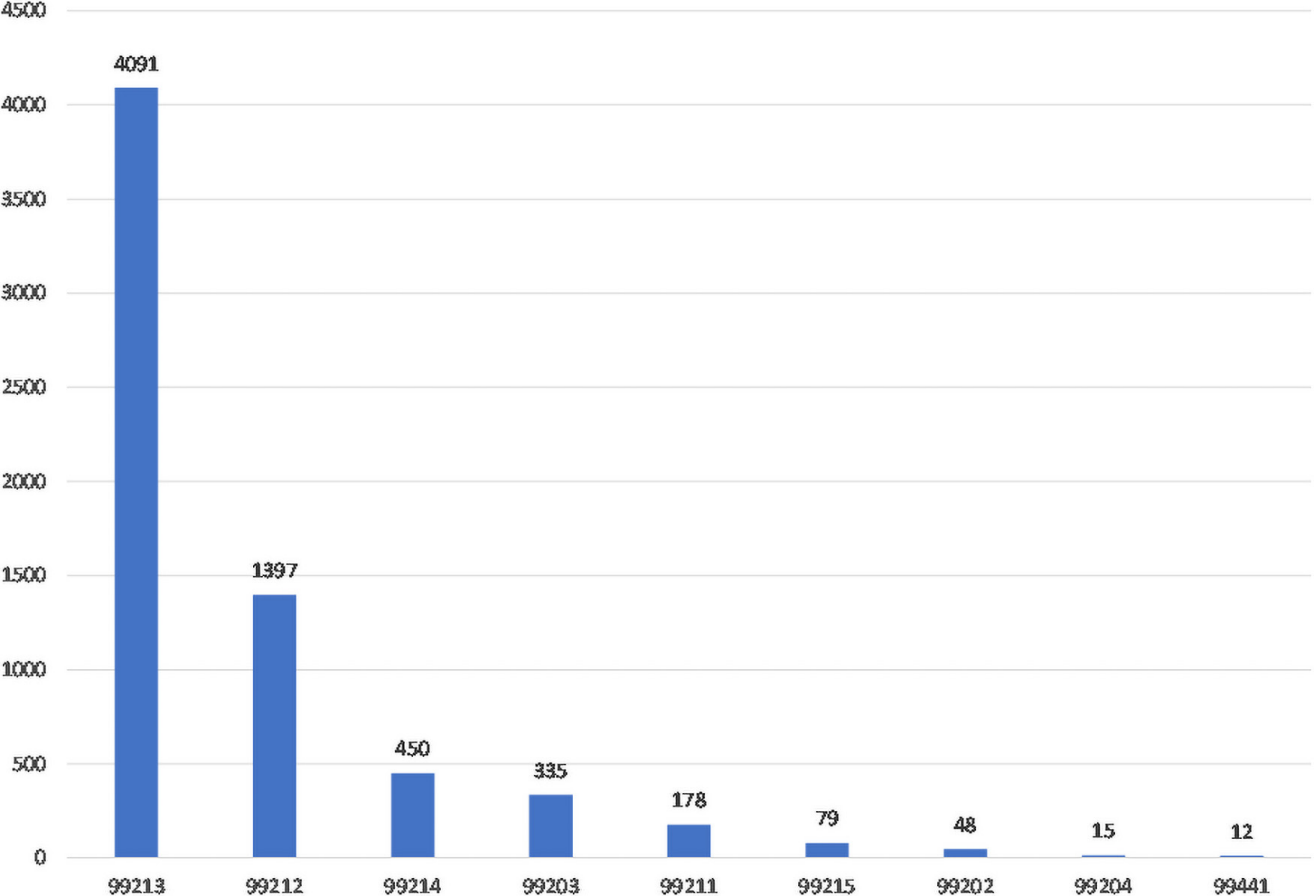
Visits by month and average wait time. Vertical axis = number of visits. Horizontal axis = month and average wait time (in black).

Operational changes were necessary in the first year to balance wait times, patient volume, and provider availability. For the first year, the service was available from 6 a.m to 12 a.m. during Monday–Friday and from 9 a.m. to 6 p.m. during Saturday–Sunday and holidays. Figure 4 outlines the main reasons that patients use the service and Figure 5 shows the wide age distribution of use. Our program serves a wider age of distribution than what previous publications have reported on DTC telemedicine programs.^2,3^ We are investigating what factors have facilitated an improved adoption among older patients.

**FIG. 4.**
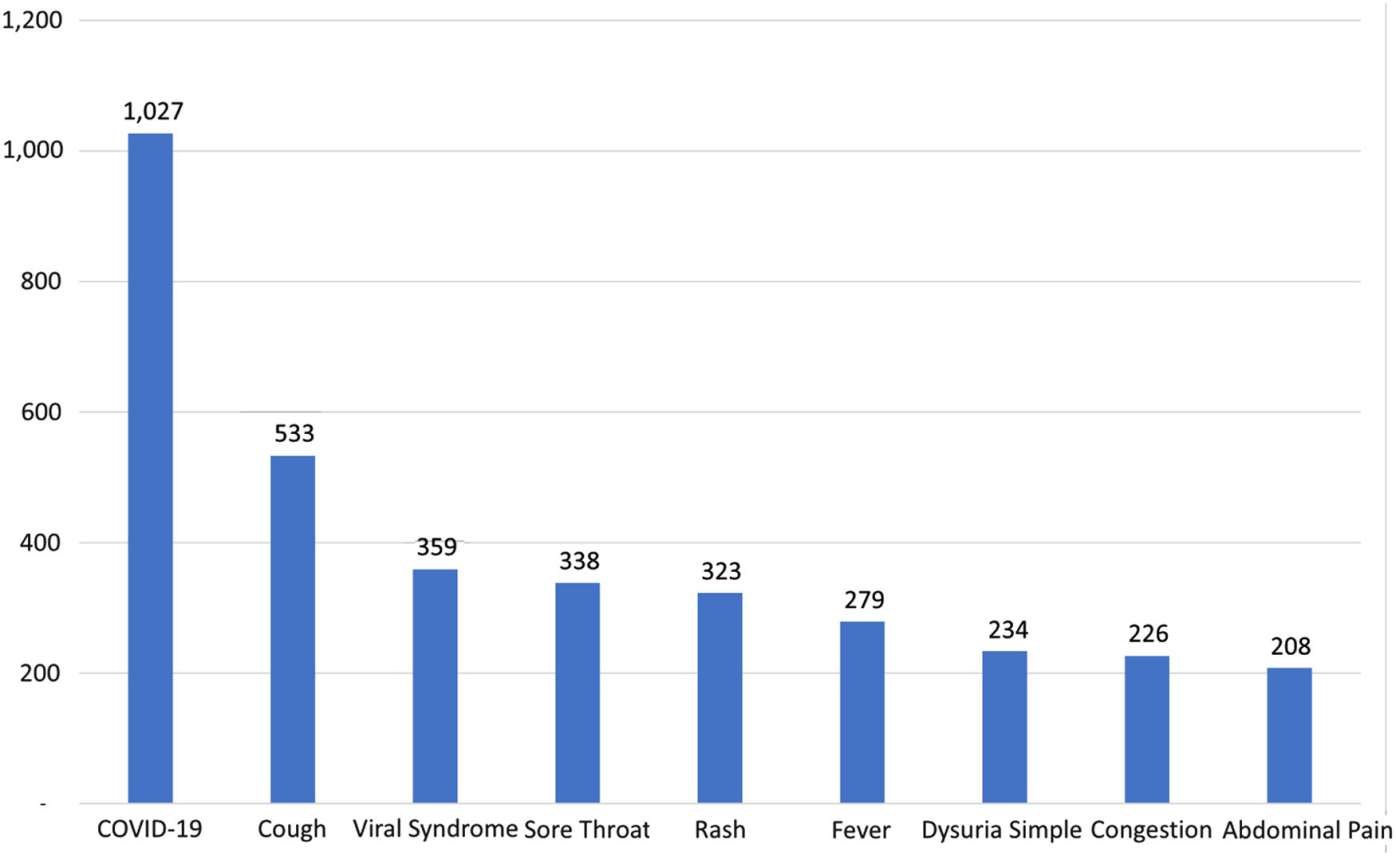
Number of visits by chief complaint. Vertical axis = number of visits. Horizontal axis = chief complaint.

**FIG. 5.**
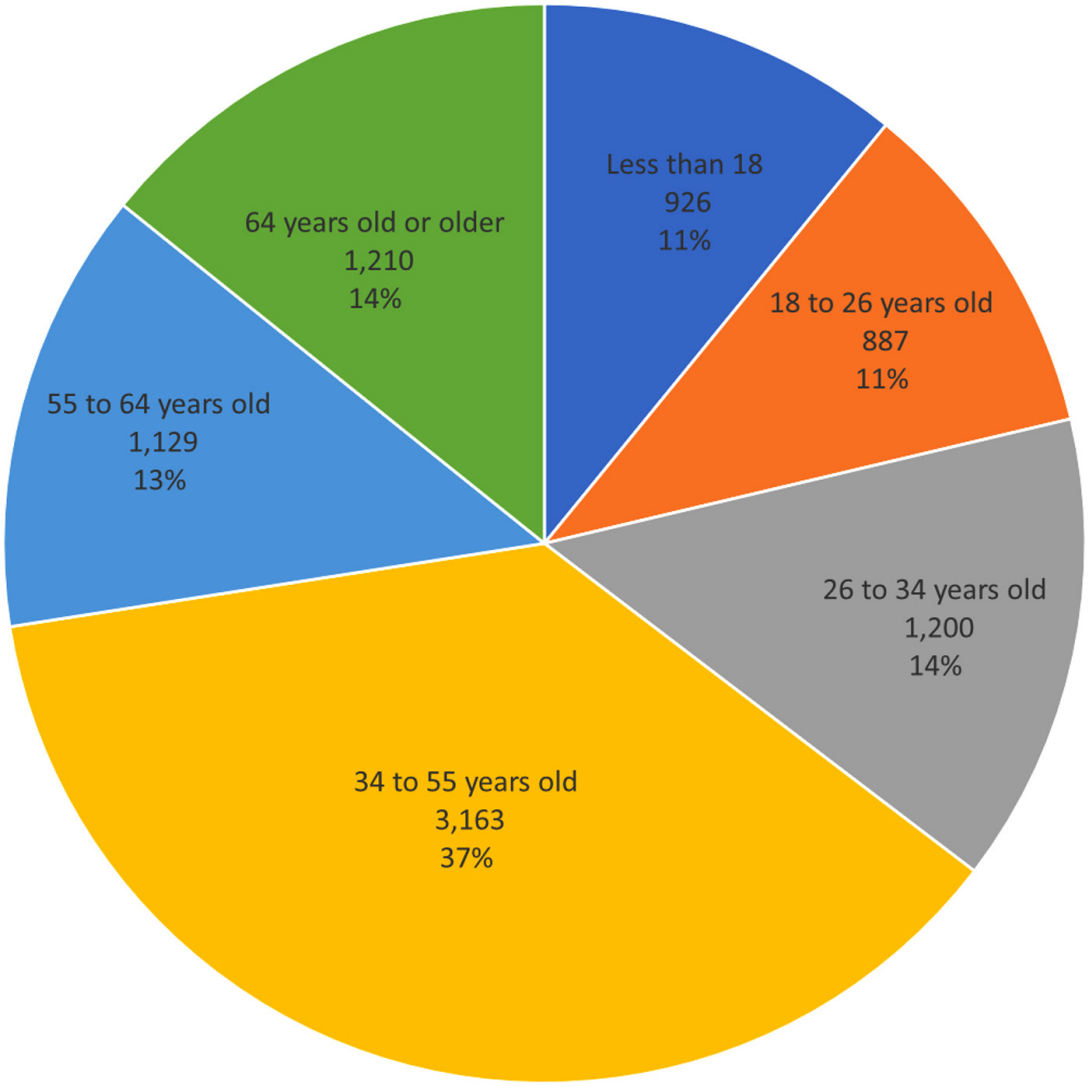
Number of visits by age. Pie chart highlighting the percentage of visits by age.

COVID-19 care is illustrative of the multiple levels of care that are achieved through our DTC academic telemedicine program. Patients who are affiliated and unaffiliated with our health system can call and discuss their symptoms and obtain testing with a turnaround time of ∼6 h for test results. If a patient appears ill, the Express Care provider will call ahead to the emergency department and facilitate a safe care plan with the internal triage team. If the patient is seen in the emergency department, a follow-up care order exists to have the patient contacted by the nurse team within 24 h to check on the patient's clinical status.

Patients were contacted after leaving the emergency department the day after discharge. If the patient was recently seen in clinic and continues to feel unwell, the Express Care provider can consult in real time with the infectious disease provider, have monoclonal antibody treatment approved, and have the nurse team coordinate outpatient treatment—in lieu of going to the emergency department. Our team is working to measure the impact of this coordination on patient–provider experience, health outcomes, and the financial impact (downstream costs and utilization of services).

What has been most rewarding is the unexpected ways in which the service has iteratively been designed to facilitate patient and provider needs. The service has become regarded an operational engine for the evolving digital infrastructure and front door of our health system. Multiple successful partnerships have occurred since its beginning to include population health, remote patient monitoring, managed care, employee health, and our cancer center.

Developing a unique model of virtual care does have a limitation. We do not have a reference point or reference to compare with—our reference has been and will continue to be the patient–provider experience. We are beginning to analyze our data and present this model as we are interested to receive feedback to improve our service and inspire other health systems to invest in creating their own.

## Abbreviations Used

DTC: direct-to-consumer
EMR: Epic Systems Corporation
